# Hyperphosphorylation-Induced Tau Oligomers

**DOI:** 10.3389/fneur.2013.00112

**Published:** 2013-08-15

**Authors:** Khalid Iqbal, Cheng-Xin Gong, Fei Liu

**Affiliations:** ^1^Department of Neurochemistry, New York State Institute for Basic Research in Developmental Disabilities, Staten Island, NY, USA

**Keywords:** microtubule associated protein tau, abnormal hyperphosphorylation of tau, O-GlcNAcylation of tau, protein phosphatase 2A, alternate splicing of tau, Alzheimer neurofibrillary degeneration, Alzheimer disease, tauopathies

## Abstract

In normal adult brain the microtubule associated protein (MAP) tau contains 2–3 phosphates per mol of the protein and at this level of phosphorylation it is a soluble cytosolic protein. The normal brain tau interacts with tubulin and promotes its assembly into microtubules and stabilizes these fibrils. In Alzheimer disease (AD) brain tau is three to fourfold hyperphosphorylated. The abnormally hyperphosphorylated tau binds to normal tau instead of the tubulin and this binding leads to the formation of tau oligomers. The tau oligomers can be sedimented at 200,000 × *g* whereas the normal tau under these conditions remains in the supernatant. The abnormally hyperphosphorylated tau is capable of sequestering not only normal tau but also MAP MAP1 and MAP2 and causing disruption of the microtubule network promoted by these proteins. Unlike Aβ and prion protein (PrP) oligomers, tau oligomerization in AD and related tauopathies is hyperphosphorylation-dependent; *in vitro* dephosphorylation of AD P-tau with protein phosphatase 2A (PP2A) inhibits and rehyperphosphorylation of the PP2A-AD P-tau with more than one combination of tau protein kinases promotes its oligomerization. In physiological assembly conditions the AD P-tau readily self-assembles into paired helical filaments. Missense tau mutations found in frontotemporal dementia apparently lead to tau oligomerization and neurofibrillary pathology by promoting its abnormal hyperphosphorylation. Dysregulation of the alternative splicing of tau that alters the 1:1 ratio of the 3-repeat: 4-repeat taus such as in Down syndrome, Pick disease, and progressive supranuclear palsy leads to the abnormal hyperphosphorylation of tau.

In Alzheimer disease (AD) the oligomer states of Aβ and tau pathologies are believed to cause the neurodegeneration. Oligomer is an intermediate stage between monomer and a large polymer. It consists of a relatively small and identifiable number of monomers, which is usually 3–10 in the case of most proteins. Unlike a polymer, if one of the monomers is removed from an oligomer, its chemical properties are altered. Protein oligomers may be formed by the polymerization of a number of monomers or the depolymerization of a large protein polymer. Protein polymerization is employed by the cell to perform several useful functions, such as neurofilaments and actin filaments serve as cytoskeleton of a neuron and maintain the cell shape. Microtubules that are polymers of tubulin facilitate axoplasmic flow, a vital function of a neuron. Some protein polymerization reactions are very efficient and almost all the protein in the cell is seen as polymers, as is the case with neurofilaments. In contrast, microtubule assembly and disassembly are extremely dynamic to meet the axoplasmic transport needs of a neuron. The oligomers of neurofilaments and microtubules are apparently very short-lived and are, to date, of no known deleterious consequence.

In AD, Aβ and, in the case of tau also in tauopathies, the protein polymerization is apparently employed as a detoxifying process to get rid of the toxic protein oligomers, which seem to stay in the diseased brain and have been isolated and studied. Tau oligomerization is increasingly being suspected as a prion-like phenomenon. This article, which is an update of our previous article on this subject (1), discusses the tau oligomers seen in AD brain and how they differ from Aβ and PrP oligomers.

In human brain tau is alternatively spliced into six isoforms and the ratio of the 3-repeat: 4-repeat protein is altered in different tauopathies. The alternative splicing of human tau pre-mRNA results in six molecular isoforms of the protein (2). These six tau isoforms differ in containing three (3R) or four (4R) microtubule binding repeats (R) of 31–32 amino acids in the carboxy-terminal half and one (1N) or two (2N) amino-terminal inserts (N) of 29 amino acids each; the extra repeat in 4R tau is the second repeat (R2) of 4R taus. This alternative splicing of tau pre-mRNA results in the expression of three 3R taus (0N3R, 1N3R, 2N3R) and three 4R taus (0N4R, 1N4R, 2N4R). The 2N4R tau is the largest size human brain tau with a total of 441 amino acids (tau_441_) in length. The smallest size tau isoform, which lacks both the two amino-terminal inserts and the extra microtubule binding repeat (0N3R; tau_352_) is the only isoform that is expressed in fetal human brain. Tau has little secondary structure; it is mostly random coil with β structure in the second and third microtubule binding repeats.

In a normal mature neuron almost all tau is bound to microtubules; tubulin is present in over 10-fold excess of tau. The concentration of tau in a neuron is ∼2 μM (3, 4) and it binds to microtubules at a kd (dissociation constant) of ∼100 nM (5). Overexpression of tau causes microtubule bundling in cultured cells. However, neither in AD nor in any tauopathy has microtubule bundling been reported.

Neurofibrillary degeneration not only is seen in AD and Down syndrome (DS) but also in a family of related neurodegenerative diseases called tauopathies. These include frontotemporal dementia with Parkinsonism linked to chromosome 17 (FTDP-17) caused by tau mutations, Pick disease, corticobasal degeneration, dementia pugilistica, and progressive supranuclear palsy. In every one of these tauopathies the neurofibrillary pathology is made up of abnormally hyperphosphorylated tau and these pathological changes in the neocortex are associated with dementia; in a large number of supranuclear palsy cases the tau pathology in the brain stem is associated with motor dysfunction.

## Oligomerization of Tau and How it Differs from That of Aβ and PrP

**Figure 1:**
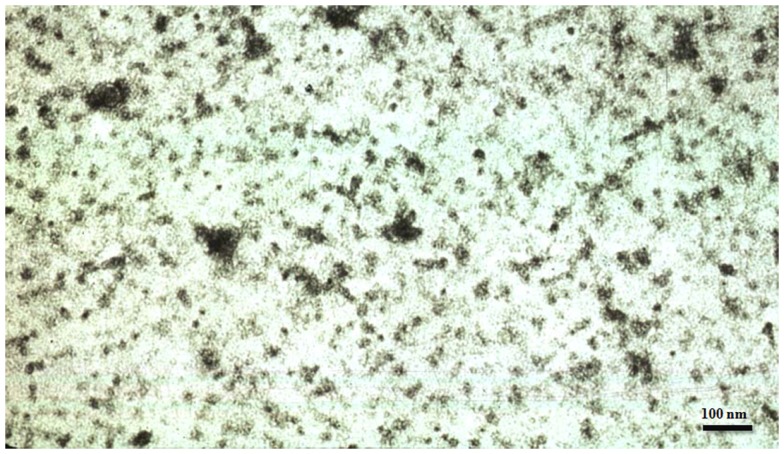
**Electron micrograph showing tau oligomers from an Alzheimer disease brain negatively stained with phosphotungstic acid**.

In 1986 we discovered that not only Alzheimer neurofibrillary tangles were made up from abnormally hyperphosphorylated tau protein (6) but also the altered tau was present in AD brain cytosol and was responsible for the inhibition of microtubule assembly (7). In subsequent studies we showed that the cytosolic AD abnormally hyperphosphorylated tau (AD P-tau) sequestered some of the normal tau and sedimented at 200,000 × *g*, whereas most of the non-hyperphosphorylated tau from the same AD brains remained in the 200,000 × *g* supernatant (8, 9). The AD P-tau showed up as globular particles by negative stained electron microscopy (Figure 1). The sedimentable AD P-tau is increasingly being referred to as the oligomeric tau or granular tau (10). *In situ* demonstration of oligomeric tau seen immunohistochemically as amorphous aggregates in the neuronal cytoplasm was described at “stage 0” tangles for the first time by Bancher et al. (11). Biochemical analysis of AD P-tau sedimented from AD brain showed that it co-sedimented some of the non-hyperphosphorylated tau, suggesting that the AD P-tau oligomers are hetero-oligomers of hyperphosphorylated and non-hyperphosphorylated tau (8). Furthermore, normal tau was found to co-aggregate with and promote the aggregation of AD P-tau into filaments (12). As much as 40% of abnormally hyperphosphorylated tau in AD brain is seen as AD P-tau (8).

Unlike normal tau which binds to tubulin and promotes its assembly into microtubules, the AD P-tau, instead of interacting with tubulin, binds to normal tau as well as MAP1 and MAP2, and causes depolymerization of microtubules (9, 12, 13). *In vitro* hyperphosphorylation of tau revealed that the oligomeric tau was an intermediate stage between monomeric and filamentous state because at 4–6 mol phosphate/mole protein it became oligomeric and microtubule-assembly inhibitory whereas further hyperphosphorylation made it polymerize into filaments. Neither the *in vitro* formed hyperphosphorylated tau filaments nor PHF isolated from AD brains had any detectable effect on tau-promoted assembly of microtubules (14–16). While normal tau promoted GTP binding to tubulin and its assembly into microtubules, the AD P-tau inhibited this activity. AD-PHF had no effect on GTP binding but on *in vitro* dephosphorylation it promoted GTP binding to tubulin (17). On dephosphorylation with protein phosphatase 2A (PP2A) the AD P-tau oligomers are converted into normal-like non-sedimentable protein that, like normal tau, promotes microtubule assembly (9, 12, 18). PP2A was also found to dissociate Alzheimer neurofibrillary tangles, releasing protein which behaved like normal tau in promoting microtubule assembly (19). Thus, the AD P-tau oligomerization is unique because it is solely induced by abnormal hyperphosphorylation and is reversible on dephosphorylation of the protein (20).

In the AD field the interest in oligomers started with the initial report of Lambert et al. (21) who showed that diffusible, non-fibrillar ligands from Aβ_1–42_ were potent central nervous system toxins. Though Aβ oligomers are toxic, in contrast to tau oligomerization, they are formed by the strong hydrophobic nature of this peptide and this process is not initiated or promoted by phosphorylation. Similarly, the PrP oligomers are formed at acidic pH and on removal of denaturants such as sodium dodecyl sulfate or salt from the protein solution (22, 23). Unlike AD P-tau and Aβ_1–42_ oligomers, the PrP filaments are the infective state and their depolymerization into oligomers results in the loss of the infectivity (24). Most recently PrP cellular has been reported to promote the Aβ oligomerization (25).

## Role of O-GlcNAcylation in Tau Oligomerization and Neurodegeneration

In addition to phosphorylation, tau is also modified by O-GlcNAcylation, a dynamic protein posttranslational modification, by which O-linked β-*N*-acetylglucosamine (O-GlcNAc) is transferred enzymatically from a UDP-GlcNAc donor to the hydroxyl group of serine or threonine residues of proteins. In contrast to glycosylation of secreted and membrane proteins, which occurs in the endoplasmic reticulum and Golgi apparatus, O-GlcNAcylation modifies nucleocytoplasmic proteins and is more like protein phosphorylation (26). O-GlcNAcylation and phosphorylation sometimes occur at identical or proximal sites of a protein and thus are reciprocal to each other. The crosstalk between O-GlcNAcylation and phosphorylation has been implicated to be essential for the control of vital cellular processes and for understanding the mechanisms of certain diseases (27, 28). O-GlcNAcylation also serves as a sensor of intracellular glucose metabolism (29), because the UDP-GlcNAc donor for O-GlcNAcylation is formed from glucose metabolism via the hexosamine biosynthetic pathway.

Tau is highly modified by O-GlcNAc, on average, with four O-GlcNAc groups per tau molecule at more than 12 serine/threonine residues (30, 31). Five O-GlcNAcylation sites (Thr123, Ser208, Ser238, Ser400, and one site at Ser409, Ser412, or Ser413) have been mapped to date (32–34). We previously demonstrated that inhibition of O-GlcNAcylation leads to hyperphosphorylation of tau in cultured cells and in rat brain slices (31). Experimental reduction of brain glucose metabolism leads to decreased O-GlcNAcylation and increased phosphorylation of tau *in vivo* (27, 35), and inhibition of protein O-GlcNAcylation induces hyperphosphorylation of tau in rat brain (27). Furthermore, we discovered that the global O-GlcNAcylation of proteins, especially of tau, is decreased, which likely results from impaired brain glucose metabolism, and that the decrease in O-GlcNAcylation correlates to hyperphosphorylation of tau in AD brain (27). Furthermore, hyperphosphorylated tau purified from AD brains contains approximately five times less O-GlcNAc than normal tau (27). Therefore, we postulate that tau pathology and neurodegeneration can be caused by impaired brain glucose metabolism via the down-regulation of tau O-GlcNAcylation in AD (27).

O-GlcNAcylation may also inhibit tau oligomerization directly. The fourth microtubule binding repeat of tau self-aggregates at a slower rate *in vitro* when it is modified by O-GlcNAc at Ser356 than the unmodified counterpart, as determined by turbidity, precipitation assay, and electron microscopy (36). A recent study showed that O-GlcNAcylation inhibits tau aggregation in rodents (37). O-GlcNAcylation also modulates proteotoxicity in *C. elegans* models of human neurodegenerative diseases (38, 39). Therefore, decreased O-GlcNAcylation may promote tau-mediated neurodegeneration through promoting tau oligomerization directly and also indirectly by inducing its abnormal hyperphosphorylation.

## Abnormal Hyperphosphorylation of Tau Causes Neurodegeneration and Cognitive Impairment

Protein phosphatase 2A accounts for ∼70% of the total tau phosphatase activity in the brain (40). A cause of the abnormal hyperphosphorylation of tau in AD and adults with DS is a decrease in the brain PP2A activity (41–43). PP2A activity is negatively regulated by two inhibitor proteins, I_1_^PP2A^ and I_2_^PP2A^ in a substrate-specific manner (44, 45). Both I_1_^PP2A^ and I_2_^PP2A^ inhibit PP2A activity toward AD hyperphosphorylated tau (46) and these inhibitors are predominantly localized in the hippocampus and the cerebellum (47). I_1_^PP2A^, which is also known as PHAP-1, is a 239 amino acid long cytoplasmic protein (48). I_2_^PP2A^, also known as SETα, PHAP-II, and TAF1β, is primarily a nuclear protein of 277 amino acids in length with an apparent molecular weight of 39 kDa on SDS-PAGE (45, 49, 50). mRNA and protein expression levels of both I_1_^PP2A^ and I_2_^PP2A^ are selectively increased in the affected areas of AD brain. I_2_^PP2A^, which is a 39 kDa and a primarily nuclear protein, is selectively cleaved at N175 into an amino-terminal (I_2NTF_) and a carboxy-terminal (I_2CTF_) fragment and translocated from the neuronal nucleus to the cytoplasm in AD brain (51). Both I_2NTF_ and I_2CTF_ interact with the PP2A catalytic subunit PP2Ac and inhibit its activity toward hyperphosphorylated tau (52). Transduction of the brains of newborn rats with adeno associated virus serotype 1 vector carrying human I_2CTF_ (53) or I_2NTF_ and I_2CTF_ transgenes was found to induce AD-like abnormal hyperphosphorylation and aggregation of tau, a loss of neuronal plasticity, and cognitive impairment in these animals at 5–12 months post-infection (54); however, no neurofibrillary tangles or Aβ plaques were detected in the brains of AAV1-I_2NTF-CTF_ rats up until 13 months. These findings suggest a deleterious role of the abnormally hyperphosphorylated oligomeric tau.

The inhibitory activity of the non-fibrillized abnormally hyperphosphorylated tau has been confirmed in yeast, drosophila, and in mouse models that express human brain tau. The expression of the longest human brain tau (2N4R tau) in yeast produces pathological phosphoepitopes, assumes a pathological conformation, and forms aggregates. These processes are modulated by yeast kinases Mds1 and Pho85, orthologs of GSK-3β and cdk5 (55, 56). In yeast the aggregation of tau increases with increasing hyperphosphorylation and the mobility in SDS-PAGE retards. The hyperphosphorylated tau isolated from the stably transfected yeast is able to assemble into filaments, and nucleate the assembly of the normal non-phosphorylated tau. These yeast studies, like those carried out previously using AD P-tau, suggest that the hyperphosphorylated tau works as a nucleation factor that initiates and promotes the aggregation of tau (12, 15).

In wild-type human tau- and mutated human tau-transgenic *Drosophila*, the accumulation of the abnormally phosphorylated tau in the absence of its fibrillization into neurofibrillary tangles leads to neurodegeneration (57). In a P301L tau inducible transgenic mouse model, cognitive improvement was observed when expression of human tau, which became abnormally hyperphosphorylated, was suppressed although neurofibrillary tangles continued to form, suggesting that the accumulation of the cytosolic abnormally hyperphosphorylated tau, and not its aggregation, was apparently involved in behavioral impairment in these animals (58). Reduction of soluble Aβ and soluble abnormally hyperphosphorylated tau, but not soluble Aβ alone, was found to ameliorate cognitive decline in 3xTg mice that express both plaque and tangle pathologies (59). Furthermore, *in vitro* dephosphorylation of neurofibrillary tangles disaggregates filaments and, as a result, the tau released behaves like normal protein in promoting microtubule assembly (19).

Hyperphosphorylation of tau, though not to the same level as in AD, is not only associated with the disease as in tauopathies, but is also employed by the neuron to down regulate its activity transiently and reversibly where required. For instance, during development the level of tubulin in the brain is at its highest, i.e., almost 33% of total cytosolic protein, which is almost 1.5-fold the critical concentration of 4 mg/ml tubulin required for its polymerization into microtubules (60). Probably to avoid microtubule bundling, the fetal tau is transiently hyperphosphorylated during development. However, the level of hyperphosphorylation of tau in fetal brain is far less than that seen in AD brain. Similarly, anesthesia and hypothermia induced by hibernation in animals induces transient hyperphosphorylation of tau (61–64). The molecular mechanism of the transient hyperphosphorylation of tau observed during development is, at present, not understood. However, during hypothermia the activity of PP2A is transiently and reversibly reduced and is believed to cause the hyperphosphorylation of tau (62, 63). In AD and DS the decrease in brain PP2A activity apparently involves different molecular mechanisms, and occurs in a non-transient and irreversible manner (41–43). It is the non-reversible nature of the abnormal hyperphosphorylation of tau in AD, DS, and related tauopathies which results in an involuntary slowing down of neuronal activity and a consequent chronic progressive neurodegeneration and its clinical phenotype, the dementia.

There is approximately as much tau in the somatodendritic compartment as in the axon (65). In the somatodendritic compartment tau is associated with rough endoplasmic reticulum and Golgi apparatus (7, 8, 66, 67). The abnormal hyperphosphorylation of tau and its accumulation in the somatodendritic compartment in AD might have been responsible for the morphological alterations of the RER and the Golgi apparatus and the abnormal N-glycosylation of tau in AD (68–71). In AD brain abnormally hyperphosphorylated tau, in addition to forming neurofibrillary tangles, is associated with granulovacuolar changes (6, 72–74). Overexpression of tau, which results in its hyperphosphorylation, has been found to induce fragmentation of Golgi both in neuronal cultures and in neurons in JNPL3 P301L tau-transgenic mice (66). In P301S tau-transgenic mice, which show abnormal hyperphosphorylation of tau, a selective decrease in mitochondria and RER has been observed (75). The chronic accumulation of the hyperphosphorylated tau as a misfolded protein in the ER could cause neurodegeneration due to protracted ER stress (76). Hyperphosphorylation of tau might also be involved in neurodegeneration through alterations of RER and Golgi and a consequent reduction in RER and mitochondria.

In addition to abnormal hyperphosphorylation, truncation of tau has been found in neurofibrillary tangles in AD and in mutated tau overexpression transgenic mouse models [e.g., (77–82)]. Of all the proteases that can cleave tau, the role of caspases has been studied the most (83, 84). Caspase 3 and caspase 6 cleave tau at D421 and D13, respectively, and treatment with Aβ can induce the D421 cleavage in cultured neurons (78, 80, 81). Truncation of tau, along with its hyperphosphorylation, promotes its aggregation into fibrils (85, 86). Although only a small fraction of tau is truncated in AD, the truncated protein can apparently recruit the full-length protein to co-aggregate with it in both tau-transgenic rat and mouse models (87, 88). To date, the bulk of the evidence suggests the soluble hyperphosphorylated tau is neurotoxic and upstream of truncation and aggregation of this protein into neurofibrillary tangles [e.g., (89, 90)].

## Role of Mutations and Alternative Splicing of Tau in Neurodegeneration

In FTDP-17 several mutations in tau co-segregate with the disease (91–93). Four of these missense mutations, G272V, P301L, V337M, and R406W, which have been most studied to date, all make tau a preferable substrate for abnormal hyperphosphorylation *in vitro* (94). Some of the tauopathies are associated with altered alternate splicing of tau. In normal human brain the 3-repeat and 4-repeat taus are expressed in 1:1 ratio.

In some of the FTDP-17 mutations, i.e., tau_K257T_ (95), tau_G272V_ (96), tau_ΔK280_ (97), tau_E10+19_, and tau_E10+29_ (98), and in Pick disease most of the tau is 3R isoforms due to the exclusion of axon 10 which codes for the second microtubule binding repeat (R_2_). In contrast, in other FTDP-17 mutations, cortical basal degeneration and progressive supranuclear palsy, most of the tau is 4R (99, 100).

How the imbalance of 3R tau/4R tau leads to neurofibrillary degeneration and dementia is currently not understood. The 4R taus bind microtubules more readily than 3R taus. Thus, a change in 3R:4R ratio of 1:1 in tauopathies results in free tau that is unbound to microtubules and free tau becomes a favorable substrate for abnormal hyperphosphorylation (101). In DS brain an increase in 3R:4R ratio combined with an extra copy of Dyrk1A, which can hyperphosphorylate tau, results in tau pathology during the fourth decade of life which is almost two decades earlier than the average age of onset of AD (102, 103).

Hyperphosphorylation by brain protein kinases induces the self-assembly of all six human brain tau isoforms into tangles of PHF/SF under physiological conditions of protein concentration, ionic strength, pH, temperature, reducing conditions, and the absence of any cofactor (9). The hyperphosphorylation of tau is catalyzed by one or more combinations of the proline-directed protein kinases (PDPKs) and non-PDPKs. Phosphorylation of tau by non-PDPKs generally primes taus for hyperphosphorylation by PDPKs (20, 104–106). Tau isoforms *in vitro* might be phosphorylated differentially. 2N4R tau is a more favorable substrate for phosphorylation by rat brain protein kinases and is phosphorylated faster and to a higher extent than 2N3R tau at Thr181, Ser199, Ser202, Thr205, Thr212, Ser214, Thr217, Thr231, Ser235, Ser262, Ser396, Ser404, and Ser422 (94). The differential phosphorylation of 3R and 4R taus involves a combination of non-PDPKs and PDPKs because, GSK-3β alone phosphorylates tau isoforms similarly (107). Pseudophosphorylation of seven GSK-3β phosphorylation sites S199, S202, T205, T231, S235, S396, and S404, affects the aggregation of tau isoforms differently; the pseudophosphorylation at these seven sites was found to enhance arachidonic acid-induced polymerization of 0N4R tau while greatly inhibiting the aggregation of the 3R isoforms (108). Thus, phosphorylation generated by the same set of kinases could be sufficient to increase the propensity of some isoforms to aggregate while reducing the aggregation of others, resulting in the differential isoform inclusion in pathological tau aggregates (108).

Aggregation of tau isoforms is affected by the type of inducer for aggregation used. Arachidonic acid induces 4R tau to polymerize to a greater extent than 3R tau (107). 0N tau requires higher concentration of arachidonic acid to get maximal polymerization. The concentration of arachidonic acid for reaching a maximal polymerization of 1N tau and 2N tau were reported to be similar, suggesting addition of exon 3 containing isoforms does not further reduce inducer concentrations needed for maximal polymerization (107). Similar results were obtained for the heparin induction of tau isoform polymerization (107). The 2N4R tau required less heparin inducer for maximal polymerization than 1N4R and 0N4R taus. Aggregation of six tau isoforms by thiazine red inducer was also reported in a tau isoform-dependent manner. Tau exons 2 and 10 were found to promote aggregation, whereas exon 3 depressed it with its efficacy dependent on the presence or absence of a fourth microtubule binding repeat (109).

Alzheimer disease P-tau sequesters normal tau, MAP1, and MAP2 and disassembles microtubules and that the dephosphorylation of AD P-tau eliminates this toxic property (9, 13). Tau isoforms bind to AD P-tau deferentially. The binding of AD P-tau to 4R tau tends to be greater than to the corresponding 3R tau and its binding to normal human recombinant tau was found to be 2N4R > 1N4R > 0N4R and 1N4R > 1N3R > 0N3R (110). AD P-tau interacts preferentially with the tau isoforms that have the amino-terminal inserts and four microtubule binding domain repeats and that hyperphosphorylation of tau appears to be sufficient to acquire AD P-tau characteristics. Thus, lack of amino-terminal inserts and extra microtubule binding domain repeat in fetal human brain might be protective from Alzheimer’s neurofibrillary degeneration.

## Conclusion

**Figure 2:**
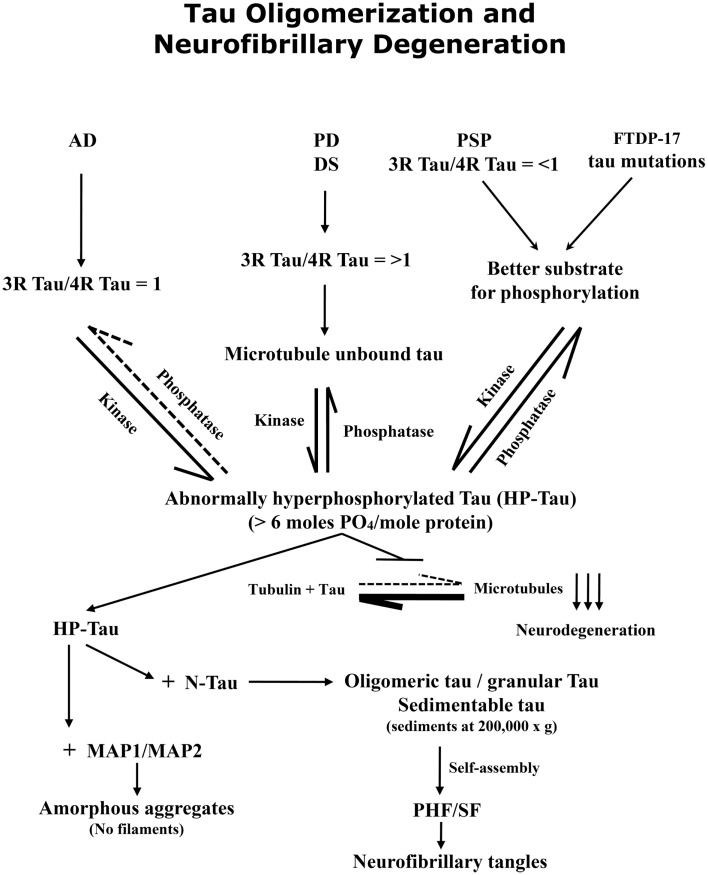
**Abnormal hyperphosphorylation of tau promotes its oligomerization and self-assembly into paired helical filaments, forming neurofibrillary tangles**. A protein phosphorylation/dephosphorylation imbalance apparently caused by a decrease in protein phosphatase 2A (PP2A) activity leads to abnormal hyperphosphorylation of tau in AD brain. The abnormally hyperphosphorylated tau binds to normal tau (and not to tubulin) and this sequestration leads to the disruption of microtubules and the formation of oligomers which can be sedimented at 200,000 × *g*; the tau oligomers show up as granular structures by negative stain electron microscopy. The abnormally hyperphosphorylated tau isolated from AD brain cytosol readily self-assembles into paired helical filaments *in vitro* under polymerizing conditions.

In conclusion, in AD and related tauopathies the abnormal hyperphosphorylation of tau promotes its oligomerization (Figure 2). The tau oligomers sequester normal tau as well as MAP1 and MAP2 and can be separated from normal tau by sedimentation at 200,000 × *g*. The abnormal hyperphosphorylation of tau seen in AD is different from the normal and from the transient hyperphosphorylation of this protein that occurs during development, anesthesia, or hypothermia. The oligomeric cytosolic AD P-tau probably causes neurodegeneration by sequestering normal MAPs and disrupting the microtubule network. Tau mutations found in frontotemporal dementia may cause neurodegeneration through promoting abnormal hyperphosphorylation of tau. AD P-tau self-assembles into PHF/SF, forming neurofibrillary tangles. Tau truncation found in AD brain promotes its self-assembly into PHF/SF. Unlike AD P-tau, the tangles neither show any detectable activity to sequester normal MAPs nor inhibit microtubule assembly. Inhibition of abnormal hyperphosphorylation of tau offers a promising therapeutic target for AD and related tauopathies. Animal models that recapitulate various disease mechanisms seen in AD and related tauopathies are no less valuable for preclinical studies for drug development than transgenic mouse and rat models in which one or more mutated human proteins are overexpressed to produce Aβ plaques and/or tau neurofibrillary tangles.

## Conflict of Interest Statement

The authors declare that the research was conducted in the absence of any commercial or financial relationships that could be construed as a potential conflict of interest.
